# Conservative Management of a Pyelovenous Fistula After a Renal Gunshot Wound

**DOI:** 10.1089/cren.2018.0090

**Published:** 2019-05-30

**Authors:** Michael Stern, Neel H. Patel, Brian Inouye, Ghalib Jibara, Judd Moul, Ariel Schulman

**Affiliations:** ^1^ Department of Urology, New York Medical College, Valhalla, New York.; ^2^ Division of Urologic Surgery, Duke University, Durham, North Carolina.; ^3^ Department of Urology, Maimonides Medical Center, Brooklyn, New York.

**Keywords:** trauma, fistula, gunshot, bullet, penetrating, renal

## Abstract

***Background:*** We report the diagnosis and management of a pyelovenous fistula that was detected 5 days after a renal gunshot wound (GSW).

***Case Presentation:*** A 16-year-old boy presented to the trauma center with a single GSW to the right flank. CT scan revealed a shattered right kidney with active contrast extravasation and ureteral discontinuity, metal fragments in the L1 vertebra, and a bullet lodged in the upper pole of the left kidney. The patient was taken for emergent exploratory laparotomy. A right nephrectomy was performed. A left retrograde pyelogram demonstrated an intact collecting system. A left Double-J stent was placed to protect against delayed thermal injury. Repeat pyelogram on postoperative day 5 revealed a pyelovenous fistula and a stent was left in place. At 6 weeks, interval pyelogram showed complete resolution of the pyelovenous fistula and the stent was removed. At 6 months the patient was asymptomatic and normotensive with an unremarkable left kidney on ultrasonography.

***Conclusion:*** Pyelovenous fistula is a rare complication of a retained bullet in the kidney. Conservative management with ureteral stenting was effective in resolving the fistula.

## Introduction and Background

Renal injury occurs in 10% of patients with abdominal trauma. Gunshot wounds (GSWs) represent a unique subset of penetrating trauma as thermal injury may take several days to manifest clinically and retained metallic fragments may lead to long-term sequelae. Unique consequences of GSWs include delayed tissue necrosis, fistulae, urinary obstruction, and bullet migration. In this report, we present a pyelovenous fistula that developed 5 days after a renal GSW that resolved after conservative management with a ureteral stent.

## Case Presentation

### Patient

A healthy 16-year-old boy was brought to the Level 1 trauma center with a GSW to the right flank. On initial evaluation, he had a heart rate of 115 and blood pressure of 120/80. Glasgow Coma Scale score was 15. Examination of the abdomen revealed a tender right lower quadrant with a bullet entry wound in the right flank and no exit wound. Foley catheter was in place without any macroscopic hematuria.

**FIG. 1.**
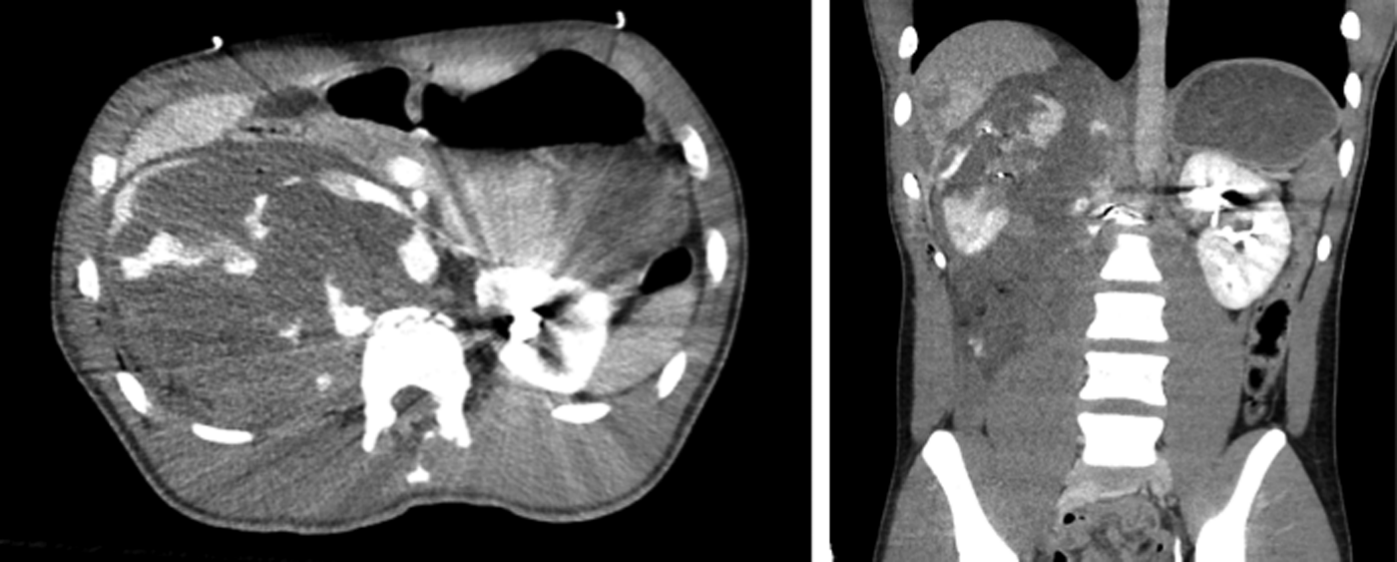
CT abdomen/pelvis showing shattered right kidney and bullet in the upper pole of the left kidney.

A CT scan of the abdomen and pelvis with intravenous contrast was performed en route to the operating room that revealed a shattered right kidney with active contrast extravasation and apparent ureteral discontinuity. There appeared to be a laceration of the posterior right hepatic lobe and a fracture of the L1 vertebral body with a paraspinal hematoma with metal fragments. A bullet was lodged in the upper pole of the left kidney (Fig. 1). The left collecting system was intact with opacification of the left ureter.

## Management

Emergent exploratory laparotomy was performed through a vertical midline incision. There was a small amount of blood in the peritoneal cavity and no enteric contents. A bulging right retroperitoneal hematoma was appreciated. A Cattel–Braasch maneuver was performed and the duodenum was kocherized with preservation of the retroperitoneal hematoma. The small intestine was mobilized and the aorta was exposed at the bifurcation and traced cranially. The left renal vein was identified on the anterior surface of the aorta and the right renal artery was identified in the interaortocaval space and encircled with a vessel loop. The anterior surface of the inferior vena cava was further exposed and the right renal vein was identified and encircled with a vessel loop.

**FIG. 2.**
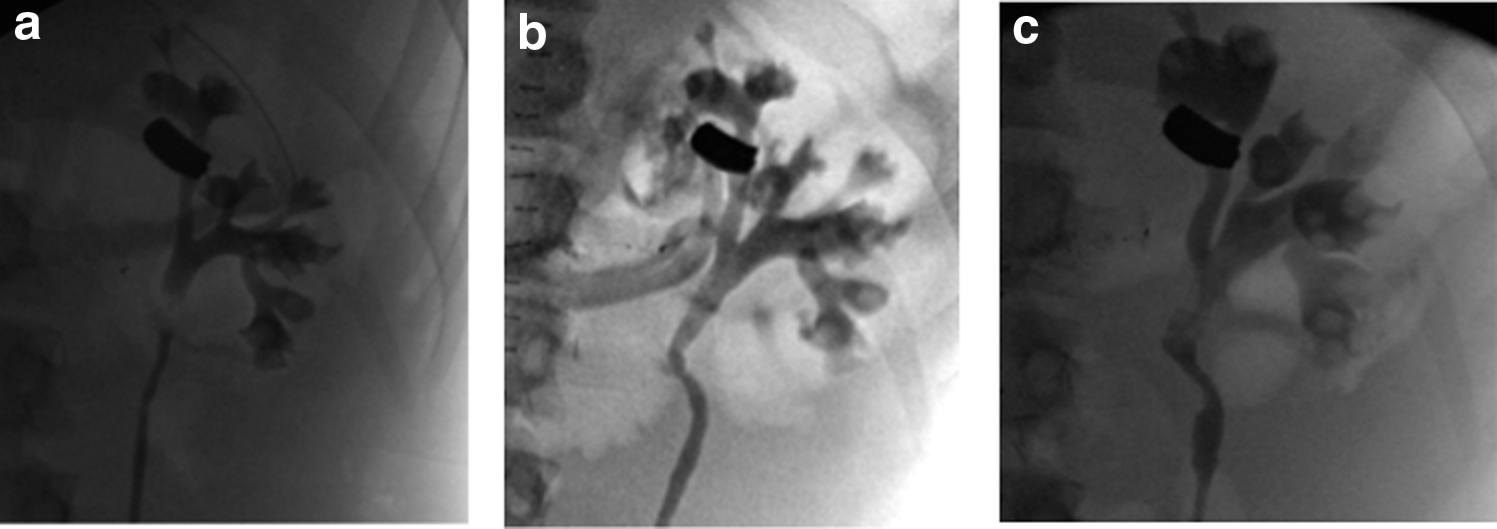
**(a)** Left RPG at time of initial exploration showing an intact urinary collecting system. **(b)** Left RPG on POD 5 showing an intact ureter and collecting system, with opacification of the left renal vein. **(c)** Left RPG on POD 45 revealed an intact collecting system with no extravasation and no pyelovenous communication. POD, postoperative day; RPG, retrograde pyelogram.

With vascular control, the right-sided hematoma was opened and a significant amount of blood was evacuated. The right kidney was shattered into multiple separate parenchymal fragments. The right renal vein and artery were ligated. The retroperitoneum was completely evacuated of residual kidney fragments and blood clots. Additional arterial bleeding on the fractured L1 vertebra was controlled with clips, fulguration, and hemostatic agents. The left retroperitoneum was not explored. After the abdominal surgery, a left retrograde pyelogram (RPG) demonstrated an intact urinary collecting system (Fig. 2a). A 4.8F × 24 cm left Double-J stent was placed to protect against delayed thermal injury.

On postoperative day (POD) 5, the patient returned to the operating room for re-evaluation of the left renal collecting system with the goal of removing the stent as he had been clinically stable and without any hematuria. RPG revealed an intact ureter and collecting system, but distinct opacification of the left renal vein after gentle injection of 20 cc of contrast demonstrating a pyelovenous fistula (Fig. 2b). The fistula was confirmed by allowing the contrast to drain and performing repeat pyelogram. A 6 × 24 Double-J stent was placed to expedite healing. Interventional radiology was consulted for consideration of angioembolization and intervention was deferred as the patient was asymptomatic and there was no arterial involvement.

The postoperative course was complicated by a right-sided fluid collection requiring drain placement in the renal fossa, and a small bowel obstruction requiring total parenteral nutrition and subsequent adhesiolysis and small bowel resection.

During the sixth postoperative week, the patient was re-evaluated by urology. He denied gross hematuria and flank pain. Repeat left RPG revealed an intact collecting system with no extravasation and no pyelovenous communication (Fig. 2c). The stent was removed. At 6-month follow-up, the patient was asymptomatic with a normal blood pressure and serum creatinine. Renal ultrasonography demonstrated an unremarkable left kidney.

## Discussion

A retained bullet fragment in the kidney is a rare sequela of a GSW, with a small number of cases reported in the literature. Reported complications include urine leak, vascular fistula, and bullet migration. Although both open and endoscopic retrieval of retained fragments has been performed, Mantica et al.^1^ recently reported conservative management of a retained bullet without any adverse effects. Management remains at the discretion of the surgeon because of the rare nature of this condition and dearth of evidence supporting any particular strategy. In this hemodynamically stable patient with an effective solitary kidney, we chose conservative management because of the risks of kidney loss associated with exploration.^2^

Pyelovenous fistulae have been noted after percutaneous nephrolithotomy, blunt trauma, and during the evaluation of gross hematuria.^3^ Several authors have reported urinary and vascular fistulae after renal GSWs, including a spino-renal fistula and arterio-caval fistula.^4^ We hypothesize that this may occur from the multifactorial effects of collecting system and vascular violation, thermal injury, and impaired healing around a foreign body. Demir et al.^3^ reported resolution of a spontaneous pyelovenous fistula with maintenance of indwelling ureteral catheter for 5 days. It is difficult to distinguish pyelovenous fistula from benign pyelovenous backflow secondary to increased pressure in the collecting system during retrograde pyelography. In this study, we favored a pyelovenous fistula on POD 5 as this was demonstrated with deliberate gentle instillation of contrast on two separate pyelograms. Furthermore, this was not present at the time of the initial injury or on the follow-up pyelography after prolonged stenting.

In this case, the patient sustained simultaneous bilateral renal injuries from a single gunshot to the right flank, including a completely shattered right kidney with significant injury to the hilum and a bullet lodged in the left kidney. Emergent right nephrectomy was performed based on CT and operative findings. The left kidney was managed expectantly because the parenchyma and collecting system were viable and intact and it now served as a solitary kidney. A left stent was placed at the initial surgery to protect against delayed thermal injury to the collecting system.

Although the pyelogram at the time of the initial injury appeared normal, a repeat study on POD 5 revealed a pyelovenous fistula. We surmise that our decision to pre-emptively stent the kidney helped protect against the evolving thermal injury. Thus, we advocate immediate stenting of renal GSWs, even with an intact collecting system, to protect against delayed blast effects. The fistula was subsequently managed with continued stenting. It is worth noting that the patient did not have gross hematuria in the days after the injury suggesting that there was venous, rather than arterial communication. We discussed the case with the interventional radiology service who did not advocate angioembolization given the absence of arterial involvement. If the patient had gross hematuria or other clinical signs of active bleeding we would have elected for angioembolization.

Although we were encouraged by the normal clinical evaluation at 6 months, we recognize that this young patient remains at risk for long-term side effects, including hypertension, renal deterioration, or stone formation around the foreign body. Thus, yearly follow-up for 5 to 10 years seems prudent.

## Conclusion

We illustrate a rare pyelovenous fistula that developed after a renal GSW with a retained bullet. The fistula occurred several days after the initial injury likely from delayed thermal injury and was treated conservatively with a ureteral stent for 6 weeks. This case supports conservative management of this rare condition, although long-term follow-up of the patient is advisable.

## Abbreviations Used

CT: computed tomography
GSW: gunshot wound
POD: postoperative day
RPG: retrograde pyelogram
